# Association Between Nitrogen Dioxide Pollution and Cause-Specific Mortality in China: Cross-Sectional Time Series Study

**DOI:** 10.2196/44648

**Published:** 2024-02-05

**Authors:** Jie Zeng, Guozhen Lin, Hang Dong, Mengmeng Li, Honglian Ruan, Jun Yang

**Affiliations:** 1 Department of Internet Medical Center Guangdong Second Provincial General Hospital Guangzhou China; 2 Guangzhou Center for Disease Control and Prevention Guangzhou China; 3 Institute of Public Health Guangzhou Medical University and Guangzhou Center for Disease Control and Prevention Guangzhou China; 4 State Key Laboratory of Oncology in South China Guangdong Provincial Clinical Research Center for Cancer Sun Yat-sen University Cancer Center Guangzhou China; 5 School of Public Health Guangzhou Medical University Guangzhou China

**Keywords:** nitrogen dioxide, cause-specific mortality, stratification effect, vulnerable subpopulations, China

## Abstract

**Background:**

Nitrogen dioxide (NO_2_) has been frequently linked to a range of diseases and associated with high rates of mortality and morbidity worldwide. However, there is limited evidence regarding the risk of NO_2_ on a spectrum of causes of mortality. Moreover, adjustment for potential confounders in NO_2_ analysis has been insufficient, and the spatial resolution of exposure assessment has been limited.

**Objective:**

This study aimed to quantitatively assess the relationship between short-term NO_2_ exposure and death from a range of causes by adjusting for potential confounders in Guangzhou, China, and determine the modifying effect of gender and age.

**Methods:**

A time series study was conducted on 413,703 deaths that occurred in Guangzhou during the period of 2010 to 2018. The causes of death were classified into 10 categories and 26 subcategories. We utilized a generalized additive model with quasi-Poisson regression analysis using a natural cubic splines function with lag structure of 0 to 4 days to estimate the potential lag effect of NO_2_ on cause-specific mortality. We estimated the percentage change in cause-specific mortality rates per 10 μg/m^3^ increase in NO_2_ levels. We stratified meteorological factors such as temperature, humidity, wind speed, and air pressure into high and low levels with the median as the critical value and analyzed the effects of NO_2_ on various death-causing diseases at those high and low levels. To further identify potentially vulnerable subpopulations, we analyzed groups stratified by gender and age.

**Results:**

A significant association existed between NO_2_ exposure and deaths from multiple causes. Each 10 μg/m^3^ increment in NO_2_ density at a lag of 0 to 4 days increased the risks of all-cause mortality by 1.73% (95% CI 1.36%-2.09%) and mortality due to nonaccidental causes, cardiovascular disease, respiratory disease, endocrine disease, and neoplasms by 1.75% (95% CI 1.38%-2.12%), 2.06% (95% CI 1.54%-2.59%), 2.32% (95% CI 1.51%-3.13%), 2.40% (95% CI 0.84%-3.98%), and 1.18% (95% CI 0.59%-1.78%), respectively. Among the 26 subcategories, mortality risk was associated with 16, including intentional self-harm, hypertensive disease, and ischemic stroke disease. Relatively higher effect estimates of NO_2_ on mortality existed for low levels of temperature, relative humidity, wind speed, and air pressure than with high levels, except a relatively higher effect estimate was present for endocrine disease at a high air pressure level. Most of the differences between subgroups were not statistically significant. The effect estimates for NO_2_ were similar by gender. There were significant differences between the age groups for mortality due to all causes, nonaccidental causes, and cardiovascular disease.

**Conclusions:**

Short-term NO_2_ exposure may increase the risk of mortality due to a spectrum of causes, especially in potentially vulnerable populations. These findings may be important for predicting and modifying guidelines for NO_2_ exposure in China.

## Introduction

Ambient air pollution is one of the greatest environmental risks to human health, with 8 million deaths reported worldwide every year [1]. Rapid industrialization in China over the last decades has significantly increased the emission of pollutants. Both short and long-term exposure to ambient air pollution increases the risk of death, years of life lost, and years of disability, thereby increasing the burden of disease [2-6]. Exposure to air pollution, including particulate matter (PM), nitrogen oxides (NO_x_), ozone, and sulfur dioxide, may increase the risk of respiratory disease through oxidative damage, which occurs via inflammatory injury and the production of reactive oxygen species [7].

Nitrogen dioxide (NO_2_) is a toxic gas that is present in traffic emissions, and chronic exposure to NO_2_ is associated with respiratory inflammation, allergies, infections, and other symptoms. The current acceptable limits of NO_2_ exposure in China are 105 ppb (200 μg/m^3^) over 1 hour and an average of 21 ppb (40 μg/m^3^) annually [8]. In recent decades, however, the concentration of NO_2_ in China has exceeded the global average level [9]. Guangzhou is the capital and largest city of Guangdong province in China, with a population of 13,964,637 [10]. According to data released by the World Health Organization [11] in 2018, the annual average concentration of NO_2_ reached 50 μg/m^3^, which exceeds air quality standards (25 μg/m^3^).

Evidence clearly demonstrates that short-term exposure to NO_2_ is related to an increased risk of mortality from different diseases, showing independent and linear trends [12-16]. For instance, a multicity analysis showed NO_2_ exposure increased the all-cause mortality rates, as well as mortality due to cardiovascular and respiratory diseases [12]. However, other studies have reported a lower mortality risk due to chronic diseases in the event of NO_2_ exposure [17]. Research on the mechanisms by which NO_2_ affects mortality has come to limited conclusions. For example, it is not yet known whether NO_2_ exposure can cause endocrine disease and neoplasms.

Regarding the effect of NO_2_ on diseases, there is less evidence for the different effects caused by stratification of meteorological factors. The significance of other meteorological risk factors, such as temperature, humidity, air pressure, and wind speed, has been less frequently explored. In addition, most of the studied disease outcomes affected by NO_2_ have included only cardiovascular and respiratory diseases. Moreover, the influence of age and gender on the association between NO_2_ and mortality is still unclear. Therefore, it is essential to fully explore predisposing diseases associated with NO_2_ exposure.

To this end, we aimed to examine the relationship between short-term NO_2_ exposure and a spectrum of causes of mortality in Guangzhou to identify the influencing factors. Our research may offer unique insights into the health effects of NO_2_ and the underlying factors and help formulate public policies to protect potentially vulnerable subgroups [18].

## Methods

### Sample and Data

The daily death count in Guangzhou during the period between 2010 and 2018 was obtained from the city registry, which included all permanent residents and is published by the Guangzhou Center for Disease Control and Prevention [19]. Using the 10th Revision of the International Classification of Diseases (ICD-10), the causes of the deaths were classified as all causes (A00-Z99), nonaccidental causes (A00-R99), cardiovascular diseases (I00-I99), neoplasms (C00-D48), endocrine diseases (D50-D89, E00-E90), nervous system diseases (G00-G99), respiratory diseases (J00-J99), digestive diseases (K00-K93), genitourinary diseases (N00-N99), and external causes (V01-Y89). These 10 groups were divided further into 26 subgroups, with each group containing at least an average number of deaths per day, ensuring that the model was convergent.

### Measures of Variables

**Figure 1:**
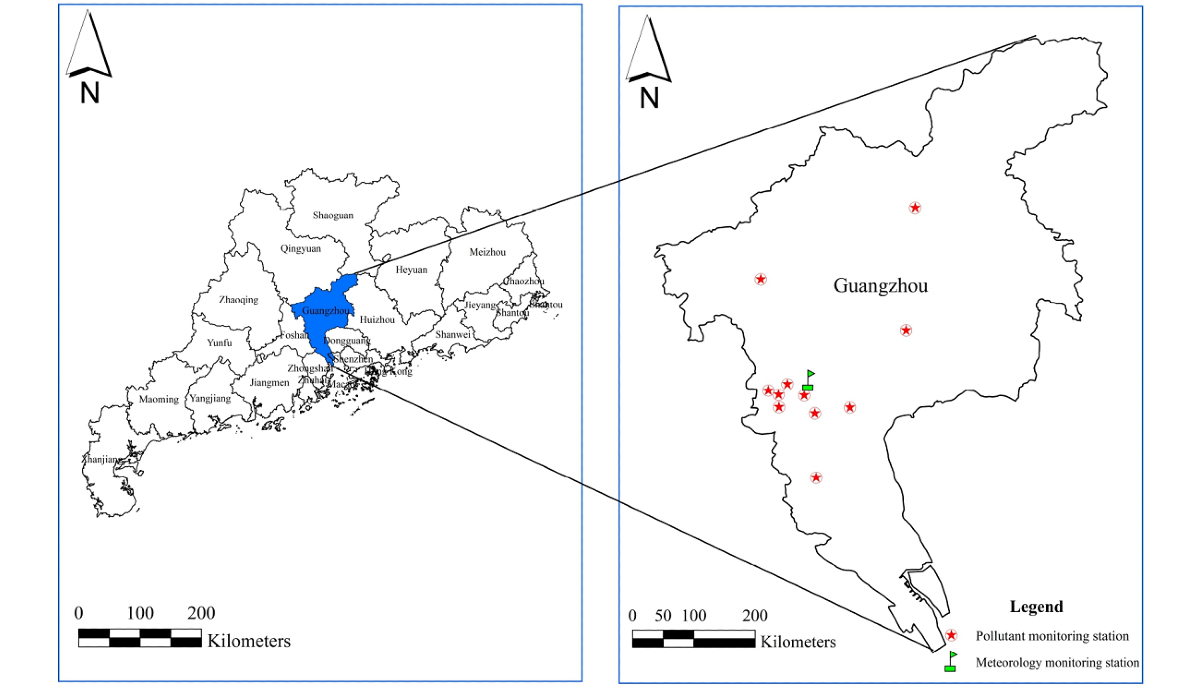
Geographic map showing the distribution of the 11 environmental monitoring stations in Guangzhou, Guangdong Province, China.

Data on the average daily level of NO_2_ from 2010 to 2018 were obtained from the Guangzhou Bureau of Environmental Protection. The data were collected from 11 fixed-point air pollution monitoring stations across the city (Figure 1). At the same time, the daily concentration of atmospheric dynamic diameter particulate matter (PM_10_ or PM_2.5_), ozone (O_3_), sulfur dioxide (SO_2_), carbon monoxide (CO), and other gaseous pollutants were obtained at the same sites and were used to adjust for potential hybrid co-pollutants during model building. Exposure levels were determined by calculating the average daily concentration level at each monitoring station. General data on the mean temperature (°C), mean relative humidity (%), mean air pressure (hPa), sunshine duration (h), wind speed (km/h), and daily air quality index were obtained from the China Meteorological Data Sharing Service System [20].

### Models and Data Analysis Procedure

A generalized additive model with Poisson regression analysis was used to determine the relationship between NO_2_ exposure and each cause of mortality after adjusting for potential covariates. A natural cubic spline function (NS) of 7 degrees of freedom (*df*) per year was used to control for long-term and seasonal trends in mortality. In the *df* adjustment of other weather factors, the NS functions of 6, 3, and 3 *df*s were used to adjust the *df*s of temperature, relative humidity, wind speed, and atmospheric pressure, respectively. The day of the week and public holidays were added to the model as indicator variables. The method and selection of model parameters have been described in a previous research analysis [21,22]. After establishing the basic model, we used an NS of 2 to 6 *df*s to investigate the relationship between NO_2_ concentration and mortality as a result of each cause of death. Based on the most likely days of lag, a moving average method was used to correlate the cumulative effects of pollutants over the defined lag period. This is consistent with a previous study [15,23] that selected an empirical maximum of 4 *dfs* for NO_2_ and used a lag model structure of 0 to 4 days to obtain an estimate of the cumulative risk of NO_2_ exposure during the first 4 days.

To identify potentially vulnerable subpopulations at a particular risk of mortality due to NO_2_ exposure on a spectrum of mortality causes, stratified analyses were conducted based on age groups (0-64 and ≥65 years) and gender (men and women) based on the causes of death that showed a significant association with NO_2_. In addition, in order to analyze the interactive health effects between meteorological factors and NO_2_, the stratification analyses were conducted based on the levels of temperature, humidity, wind speed, and air pressure, with their median values as the cutoff. The difference between the 2 groups was analyzed using a *z* test, as follows [24]:

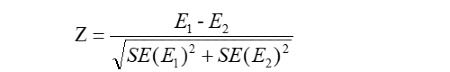



where *E_1_* and *E_2_* are the logarithms of risk ratios for 2 categories, such as men and women or 0-64 years and ≥65 years, and *SE* (*E_1_*) and *SE* (*E_2_*) were used to indicate the relevant standard error [25,26].

Multiple sensitivity analyses were conducted to evaluate the robustness of the model and parameter validation estimates. The *df*s for the time variable were set as 3 years to 7 years, and the *df*s for the weather variable were set as 3 to 6. To adjust for the mixing effect of temperature during longer lag days, 2-dimensional, cross-basis NSs and 4 *df*s were used for temperature and lag dimensions, respectively, and a maximum lag of 21 days was used for the distributed lag nonlinear model (DLNM) [27]. Dual pollutant models were constructed to verify the robustness of the main model after adjusting for confounders by adding another air pollutant (O_3_, PM_2.5_, PM_10_, CO, SO_2_) to the main model.

All statistical analyses were performed using the mgcv and dlnm packages of R software (version 3.4.1) by major models [28]. The change in mortality was calculated for every 10 μg/m^3^ increase in NO_2_ grade. All statistical results are presented as 2-sided values, while a P value <.05 was considered to indicate statistical significance.

### Ethics Approval

Ethics approval was not required for secondary analysis of the anonymous data in this study.

## Results

The average annual temperature, humidity, daily NO_2_ concentrations, and number of cause-specific deaths are summarized in Table 1. The Spearman correlation between air pollution and weather conditions is shown in Figure S1 in Multimedia Appendix 1. The correlation coefficients between NO_2_ and other constituents ranged from 0.1 to 0.94. Temperature and wind speed were negatively correlated with NO_2_, while NO_2_ was negatively correlated with other meteorological factors and air pollutants.

**Table 1:** Summary statistics of environmental monitoring and daily death counts during 2010 to 2018 in Guangzhou, China.

Variable			Mean (SD)
**Air pollutant concentrations**			
	NO_2_ (μg/m^3^)	47 (19)	
	Particulate matter (PM)_2.5_ (μg/m^3^)	38 (22)	
	SO_2_ (μg/m^3^)	18 (12)	
	O_3_ (μg/m^3^)	82 (47)	
	CO (mg/m^3^)	0.9 (0.2)	
	Air quality index	72 (31)	
**Weather conditions**			
	Temperature (°C)	22 (6)	
	Relative humidity (%)	79 (11)	
	Air pressure (hPa)	1007 (56)	
	Wind speed (km/h)	22 (11)	
**Daily numbers of deaths**			
	All causes	126 (27)	
	Nonaccidental causes	119 (26)	
	Circulatory disease	49 (14)	
	Respiratory disease	19 (7)	
	Digestive disease	4 (2)	
	Nervous disease	1 (1)	
	Genitourinary disease	2 (1)	
	External causes	7 (3)	
	Endocrine disease	5 (3)	
	Neoplasms	35 (8)	

The average annual NO_2_ level was 47 μg/m^3^. During the study period of 2010 to 2018, 413,703 all-cause deaths were recorded in Guangzhou, with 126 deaths occurring daily, on average. The majority (391,543/413,703, 94.6%) of the deaths were due to nonaccidental causes. Other causes included cardiovascular diseases (160,226/413,703, 38.7%), respiratory diseases (63,290/413,703, 15.3%), digestive diseases (12,787/413,703, 3.1%), nervous system diseases (3659/413,703, 0.9%), genitourinary diseases (5314/413,703, 1.3%), external causes (22,147/413,703, 5.4%), endocrine diseases (14,972/413,703, 3.6%), and neoplasms (115,703/413,703, 28%; Table S1 in Multimedia Appendix 1).

**Figure 2:**
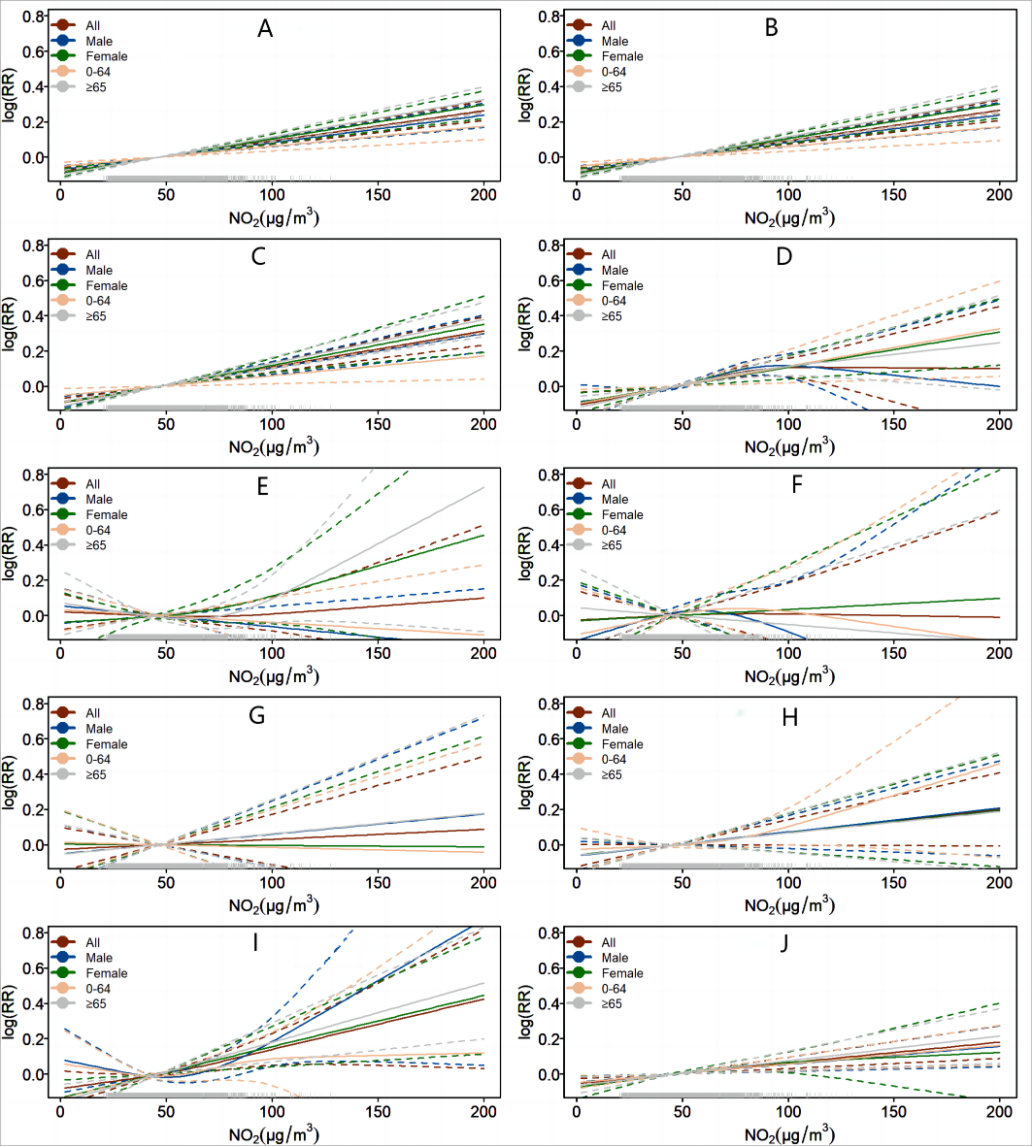
The dose-effect of the curves of NO_2_ and mortality as a result of (A) all causes, (B) nonaccidental causes, (C) cardiovascular diseases, (D) respiratory diseases, (E) digestive diseases, (F) nervous system diseases, (G) genitourinary diseases, (H) external causes, (I) endocrine diseases, and (J) neoplasms at 7 degrees of freedom with a lag of 0 to 4 days, stratified by gender and age (years).

The association between NO_2_ exposure and mortality due to specific causes over 6 days (lags 0 to 6) and stratified by gender and age are shown in Table 2 and Figure S2 in Multimedia Appendix 1. For all-cause mortality, the highest risk of death due to NO_2_ exposure was observed at the lag of 2 or 3 days and was usually limited to 4 days. Effect estimates of NO_2_ were stronger for women and older subgroups for different diseases before the lag of 4 days, and the effects stabilized thereafter. The mortality risk increased linearly with NO_2_ concentrations at a lag of 0 to 4 days (Figure 2). Finally, we examined the stability of the model by using lag 0 days for NO_2_ and observed a similar linear relationship between NO_2_ and mortality (Figure S4 in Multimedia Appendix 1). The results of the generalized Poisson regression are shown in Table S2 in Multimedia Appendix 1. The goodness of fit index *R*^2^ of the model was 0.69, which meant that the model was well constructed.

**Table 2:** The relative risk of death resulting from the different causes associated with an increase of 10 μg/m^3^ in NO_2_ levels with a 1-day delay (lag days of 0, 1, 2, 3, 4, 5, and 6) for each gender and age group.

Causes of death		All, % change (95% CI)	Gender, % change (95% CI)		Age (years), % change (95% CI)	
			Male	Female	0-64	≥65
**All causes**						
	Lag 0	1.009 (1.006-1.011)	1.009 (1.005-1.012)	1.009 (1.005-1.012)	1.007 (1.004-1.010)	1.010 (1.006-1.013)
	Lag 1	1.011 (1.008-1.014)	1.010 (1.007-1.013)	1.013 (1.010-1.017)	1.007 (1.004-1.010)	1.014 (1.011-1.017)
	Lag 2	1.010 (1.007-1.013)	1.009 (1.006-1.012)	1.012 (1.009-1.016)	1.006 (1.002-1.009)	1.014 (1.010-1.017)
	Lag 3	1.007 (1.005-1.010)	1.007 (1.003-1.010)	1.008 (1.004-1.011)	1.005 (1.001-1.008)	1.009 (1.006-1.012)
	Lag 4	1.003 (1.001-1.007)	1.004 (1.001-1.007)	1.004 (1.000-1.007)	1.003 (1.000-1.006)	1.005 (1.001-1.008)
	Lag 5	1.001 (0.998-1.003)	1.001 (0.998-1.005)	0.999 (0.996-1.003)	1.000 (0.996-1.003)	1.001 (0.998-1.004)
	Lag 6	0.997 (0.995-1.999)	0.997 (0.994-1.001)	0.997 (0.993-1.000)	0.997 (0.994-1.000)	0.997 (0.994-1.001)
**Nonaccidental causes**						
	Lag 0	1.008 (1.006-1.011)	1.009 (1.005-1.012)	1.008 (1.004-1.012)	1.007 (1.003-1.010)	1.009 (1.006-1.013)
	Lag 1	1.011 (1.009-1.014)	1.010 (1.007-1.013)	1.013 (1.010-1.017)	1.007 (1.003-1.010)	1.014 (1.011-1.017)
	Lag 2	1.010 (1.008-1.013)	1.008 (1.005-1.012)	1.013 (1.010-1.017)	1.005 (1.002-1.009)	1.014 (1.011-1.017)
	Lag 3	1.007 (1.005-1.010)	1.007 (1.003-1.010)	1.008 (1.005-1.012)	1.005 (1.001-1.008)	1.009 (1.006-1.012)
	Lag 4	1.004 (1.002-1.007)	1.004 (1.001-1.007)	1.004 (1.000-1.008)	1.003 (1.000-1.007)	1.005 (1.001-1.008)
	Lag 5	1.001 (0.998-1.003)	1.001 (0.998-1.005)	1.000 (0.996-1.003)	1.000 (0.996-1.004)	1.001 (0.998-1.005)
	Lag 6	0.997 (0.995-1.000)	0.997 (0.994-1.001)	0.997 (0.993-1.000)	0.997 (0.994-1.000)	0.997 (0.994-1.000)
**Circulatory diseases**						
	Lag 0	1.009 (1.005-1.012)	1.008 (1.004-1.013)	1.009 (1.004-1.014)	1.008 (1.002-1.014)	1.009 (1.004-1.013)
	Lag 1	1.013 (1.009-1.016)	1.011 (1.004-1.013)	1.014 (1.004-1.014)	1.006 (1.000-1.012)	1.016 (1.004-1.013)
	Lag 2	1.012 (1.008-1.016)	1.009 (1.004-1.014)	1.015 (1.004-1.014)	1.005 (1.000-1.012)	1.015 (1.011-1.020)
	Lag 3	1.009 (1.006-1.013)	1.010 (1.005-1.014)	1.009 (1.004-1.014)	1.004 (0.998-1.010)	1.012 (1.007-1.016)
	Lag 4	1.007 (1.003-1.011)	1.009 (1.004-1.014)	1.005 (1.000-1.010)	1.004 (0.998-1.010)	1.008 (1.004-1.013)
	Lag 5	1.003 (0.999-1.007)	1.004 (1.004-1.014)	1.001 (0.996-1.006)	1.002 (0.996-1.008)	1.003 (0.999-1.008)
	Lag 6	0.997 (0.993-1.000)	0.997 (0.992-1.001)	0.997 (0.996-1.006)	0.996 (0.991-1.002)	0.997 (0.993-1.001)
**Respiratory diseases**						
	Lag 0	1.010 (1.004-1.015)	1.012 (1.005-1.020)	1.006 (0.997-1.014)	1.017 (1.006-1.030)	1.008 (1.002-1.014)
	Lag 1	1.015 (1.010-1.021)	1.015 (1.008-1.022)	1.017 (1.008-1.025)	1.013 (1.001-1.025)	1.016 (1.010-1.022)
	Lag 2	1.017 (1.011-1.022)	1.015 (1.008-1.022)	1.019 (1.011-1.028)	1.010 (0.998-1.022)	1.018 (1.012-1.024)
	Lag 3	1.008 (1.003-1.014)	1.010 (1.003-1.017)	1.005 (0.997-1.014)	1.003 (0.991-1.015)	1.009 (1.003-1.016)
	Lag 4	1.005 (0.999-1.011)	1.008 (1.001-1.015)	1.001 (0.992-1.009)	1.010 (0.998-1.022)	1.004 (0.998-1.010)
	Lag 5	1.001 (0.996-1.007)	1.001 (0.994-1.008)	1.002 (0.993-1.010)	1.010 (0.997-1.022)	0.999 (0.993-1.005)
	Lag 6	0.997 (0.992-1.003)	0.996 (0.989-1.003)	1.000 (0.991-1.008)	1.001 (0.989-1.013)	0.996 (0.990-1.003)
**Digestive diseases**						
	Lag 0	1.004 (0.992-1.015)	0.997 (0.982-1.012)	1.014 (0.996-1.033)	0.996 (0.979-1.014)	1.010 (0.995-1.025)
	Lag 1	1.003 (0.992-1.015)	0.997 (0.983-1.012)	1.010 (0.992-1.029)	0.996 (0.979-1.013)	1.008 (0.993-1.023)
	Lag 2	0.999 (0.988-1.010)	0.996 (0.981-1.010)	1.003 (0.985-1.022)	0.993 (0.976-1.011)	1.003 (0.988-1.018)
	Lag 3	0.998 (0.987-1.009)	0.997 (0.982-1.011)	1.000 (0.981-1.019)	1.001 (0.984-1.019)	0.996 (0.981-1.011)
	Lag 4	0.997 (0.985-1.008)	0.990 (0.975-1.005)	1.008 (0.989-1.027)	0.999 (0.982-1.017)	0.995 (0.980-1.011)
	Lag 5	0.991 (0.980-1.003)	0.988 (0.973-1.003)	0.997 (0.978-1.016)	0.991 (0.974-1.009)	0.992 (0.976-1.008)
	Lag 6	0.995 (0.980-1.003)	0.995 (0.981-1.010)	0.995 (0.976-1.013)	0.997 (0.980-1.014)	0.994 (0.979-1.009)
**Nervous diseases**						
	Lag 0	0.995 (0.973-1.017)	0.986 (0.957-1.016)	1.005 (0.972-1.038)	0.998 (0.969-1.028)	0.990 (0.958-1.023)
	Lag 1	0.992 (0.971-1.014)	0.982 (0.953-1.012)	1.004 (0.973-1.037)	0.999 (0.971-1.028)	0.983 (0.952-1.016)
	Lag 2	0.999 (0.978-1.021)	0.995 (0.966-1.025)	1.004 (0.973-1.037)	0.995 (0.967-1.024)	1.004 (0.972-1.037)
	Lag 3	1.010 (0.988-1.032)	1.015 (0.985-1.045)	1.005 (0.973-1.038)	1.006 (0.978-1.036)	1.014 (0.981-1.047)
	Lag 4	1.009 (0.987-1.032)	1.025 (0.995-1.057)	0.991 (0.958-1.025)	1.027 (0.997-1.057)	0.985 (0.952-1.020)
	Lag 5	0.985 (0.963-1.007)	0.995 (0.965-1.026)	0.973 (0.940-1.006)	1.010 (0.981-1.039)	0.951 (0.918-0.984)
	Lag 6	0.980 (0.959-1.002)	0.977 (0.949-1.007)	0.983 (0.951-1.016)	1.004 (0.976-1.033)	0.947 (0.916-0.980)
**Genitourinary diseases**						
	Lag 0	1.009 (0.991-1.028)	1.008 (0.984-1.032)	1.012 (0.985-1.040)	1.011 (0.984-1.038)	1.008 (0.984-1.033)
	Lag 1	1.007 (0.989-1.025)	1.010 (0.986-1.034)	1.004 (0.977-1.032)	1.005 (0.979-1.032)	1.008 (0.984-1.033)
	Lag 2	1.004 (0.986-1.022)	1.018 (0.995-1.043)	0.986 (0.959-1.013)	1.006 (0.979-1.033)	1.002 (0.977-1.027)
	Lag 3	0.998 (0.979-1.016)	1.005 (0.981-1.030)	0.989 (0.961-1.017)	0.995 (0.968-1.022)	0.999 (0.975-1.025)
	Lag 4	0.994 (0.976-1.013)	0.986 (0.961-1.011)	1.005 (0.977-1.034)	0.976 (0.949-1.004)	1.009 (0.983-1.035)
	Lag 5	0.991 (0.973-1.010)	0.986 (0.962-1.011)	0.998 (0.970-1.027)	0.978 (0.951-1.006)	1.003 (0.977-1.029)
	Lag 6	0.988 (0.970-1.007)	0.995 (0.971-1.019)	0.980 (0.953-1.008)	0.989 (0.962-1.016)	0.987 (0.963-1.013)
**External causes**						
	Lag 0	1.013 (1.004-1.022)	1.008 (0.996-1.020)	1.020 (1.006-1.034)	1.012 (1.000-1.024)	1.014 (1.000-1.029)
	Lag 1	1.010 (1.001-1.020)	1.008 (0.996-1.019)	1.014 (1.000-1.029)	1.009 (0.998-1.021)	1.012 (0.998-1.027)
	Lag 2	1.006 (0.996-1.015)	1.010 (0.998-1.022)	0.999 (0.985-1.013)	1.009 (0.997-1.021)	1.001 (0.986-1.015)
	Lag 3	1.002 (0.993-1.012)	1.005 (0.993-1.017)	0.998 (0.984-1.012)	1.004 (0.992-1.016)	1.000 (0.985-1.014)
	Lag 4	1.001 (0.991-1.010)	1.001 (0.989-1.014)	1.000 (0.985-1.014)	1.000 (0.988-1.012)	1.003 (0.988-1.018)
	Lag 5	0.998 (0.988-1.007)	1.002 (0.990-1.014)	0.991 (0.977-1.006)	0.999 (0.987-1.011)	0.996 (0.981-1.011)
	Lag 6	0.998 (0.989-1.007)	0.999 (0.987-1.011)	0.996 (0.982-1.010)	0.996 (0.984-1.007)	1.001 (0.986-1.015)
**Endocrine diseases**						
	Lag 0	1.011 (1.000-1.022)	1.006 (0.991-1.022)	1.015 (1.000-1.030)	1.003 (0.986-1.019)	1.017 (1.003-1.031)
	Lag 1	1.020 (1.009-1.030)	1.017 (1.002-1.032)	1.022 (1.008-1.037)	1.010 (0.994-1.026)	1.027 (1.013-1.041)
	Lag 2	1.020 (1.009-1.031)	1.019 (1.003-1.034)	1.021 (1.007-1.036)	1.012 (0.996-1.029)	1.026 (1.011-1.040)
	Lag 3	1.007 (0.997-1.018)	1.003 (0.987-1.019)	1.011 (0.996-1.026)	1.003 (0.987-1.020)	1.010 (0.996-1.025)
	Lag 4	0.998 (0.987-1.010)	0.996 (0.980-1.012)	1.001 (0.986-1.016)	0.995 (0.978-1.012)	1.001 (0.986-1.016)
	Lag 5	0.997 (0.986-1.008)	0.996 (0.980-1.013)	0.997 (0.982-1.012)	0.992 (0.975-1.009)	1.000 (0.986-1.015)
	Lag 6	0.988 (0.977-0.999)	0.981 (0.966-0.997)	0.994 (0.979-1.008)	0.992 (0.976-1.009)	0.985 (0.971-1.000)
**Neoplasms**						
	Lag 0	1.006 (1.002-1.010)	1.008 (1.002-1.013)	1.003 (0.996-1.010)	1.003 (0.998-1.008)	1.010 (1.004-1.017)
	Lag 1	1.007 (1.003-1.011)	1.007 (1.002-1.012)	1.007 (1.000-1.013)	1.007 (1.002-1.012)	1.008 (1.001-1.015)
	Lag 2	1.007 (1.003-1.011)	1.007 (1.002-1.012)	1.007 (1.000-1.013)	1.006 (1.001-1.011)	1.008 (1.001-1.015)
	Lag 3	1.006 (1.002-1.010)	1.003 (0.998-1.008)	1.011 (1.004-1.018)	1.006 (1.001-1.011)	1.006 (0.999-1.013)
	Lag 4	1.002 (0.998-1.006)	0.999 (0.994-1.005)	1.006 (1.000-1.013)	1.003 (0.998-1.008)	1.001 (0.994-1.008)
	Lag 5	1.000 (0.996-1.005)	1.001 (0.996-1.006)	0.999 (0.992-1.006)	1.000 (0.995-1.005)	1.001 (0.994-1.009)
	Lag 6	1.000 (0.996-1.004)	1.001 (0.996-1.007)	0.999 (0.992-1.005)	0.999 (0.994-1.004)	1.004 (0.997-1.010)

Among 10 categories, for every 10 μg/m^3^ increment in NO_2_ at a lag of 0 to 4 days, the risks of mortality increased due to all causes by 1.73% (95% CI 1.36%-2.09%) and due to nonaccidental causes, cardiovascular diseases, respiratory diseases, endocrine diseases, and neoplasms by 1.75% (95% CI 1.38%-2.12%), 2.06% (95% CI 1.54%-2.59%), 2.32% (95% CI 1.51%-3.13%), 2.40% (95% CI 0.84%-3.98%), and 1.18% (95% CI 0.59%-1.78%), respectively. On the other hand, NO_2_ did not significantly impact deaths due to digestive diseases, genitourinary diseases, nervous diseases, and external causes (Table 3 and Figure S3 in Multimedia Appendix 1).

**Table 3:** Percentage increase in mortality as a result of different diseases per 10 μg/m^3^ increase in NO_2_ with a lag of 0 to 4 days in Guangzhou, China, stratified by gender and age groups.

Causes of death	All, % change (95% CI)	Gender, % change (95% CI)		Age (years), % change (95% CI)	
		Male	Female	0-64	≥65
All causes	1.73 (1.36 to 2.09)	1.56 (1.11 to 2.00)	1.95 (1.45 to 2.46)	1.15 (0.66 to 1.63)	2.15 (1.68 to 2.62)
Nonaccidental causes	1.75 (1.38 to 2.12)	1.57 (1.12 to 2.03)	1.98 (1.47 to 2.50)	1.13 (0.62 to 1.63)	2.18 (1.71 to 2.66)
Cardiovascular diseases	2.06 (1.54 to 2.59)	1.97 (1.29 to 2.65)	2.17 (1.46 to 2.90)	1.13 (0.29 to 1.98)	2.50 (1.87 to 3.12)
Chronic rheumatic heart diseases	2.32 (1.56 to 3.08)	2.07 (1.05 to 3.10)	2.62 (1.55 to 3.71)	1.62 (0.37 to 2.88)	2.68 (1.76 to 3.60)
Hypertensive diseases	3.39 (1.80 to 4.99)	2.79 (0.50 to 5.13)	3.90 (1.75 to 6.10)	1.88 (–1.15 to 5.01)	3.88 (2.08 to 5.71)
Ischemic heart diseases	1.46 (0.70 to 2.23)	1.61 (0.56 to 2.66)	1.32 (0.26 to 2.40)	0.59 (–0.74 to 1.93)	1.82 (0.91 to 2.73)
Acute ischemic heart disease	1.27 (0.20 to 2.34)	1.71 (0.30 to 3.13)	0.71 (–0.85 to 2.30)	0.84 (–0.84 to 2.56)	1.51 (0.18 to 2.87)
Acute myocardial infarction	1.23 (0.13 to 2.34)	1.69 (0.24 to 3.16)	0.65 (–0.98 to 2.30)	1.05 (–0.70 to 2.82)	1.33 (–0.05 to 2.73)
Myocardial infarction	1.23 (0.12 to 2.34)	1.67 (0.22 to 3.14)	0.66 (–0.97 to 2.31)	1.04 (–0.70 to 2.82)	1.33 (–0.05 to 2.73)
Chronic ischemic heart disease	1.65 (0.60 to 2.72)	1.54 (0.02 to 3.07)	1.77 (0.33 to 3.23)	0.21 (–1.99 to 2.45)	2.05 (0.85 to 3.26)
Other forms of heart disease	–0.61 (–3.09 to 1.93)	–0.66 (–3.97 to 2.76)	–0.59 (–4.34 to 3.30)	–1.28 (–4.81 to 2.39)	0.04 (–3.41 to 3.62)
Cerebrovascular diseases	2.48 (1.70 to 3.27)	2.21 (1.16 to 3.27)	2.80 (1.69 to 3.91)	1.64 (0.35 to 2.94)	2.89 (1.95 to 3.84)
Stroke	2.48 (1.55 to 3.42)	2.30 (1.03 to 3.58)	2.71 (1.35 to 4.08)	1.47 (–0.01 to 2.97)	3.07 (1.90 to 4.25)
Intracerebral hemorrhagic stroke	1.75 (0.21 to 3.30)	2.18 (0.21 to 4.18)	1.19 (–1.18 to 3.61)	1.23 (–0.90 to 3.40)	2.18 (0.06 to 4.35)
Ischemic stroke	2.84 (1.35 to 4.35)	2.35 (0.30 to 4.43)	3.35 (1.24 to 5.50)	0.95 (–1.72 to 3.69)	3.60 (1.85 to 5.39)
Arteries, arterioles, and capillaries	0.27 (–3.75 to 4.45)	0.44 (–4.40 to 5.53)	–0.29 (–7.17 to 7.09)	2.04 (–3.73 to 8.16)	–1.24 (–6.66 to 4.50)
Respiratory diseases	2.32 (1.51 to 3.13)	2.53 (1.50 to 3.57)	2.03 (0.82 to 3.27)	2.15 (0.41 to 3.92)	2.36 (1.46 to 3.27)
Influenza and pneumonia	2.47 (1.35 to 3.60)	2.72 (1.20 to 4.27)	2.19 (0.62 to 3.80)	2.50 (0.06 to 5.00)	2.45 (1.21 to 3.71)
Chronic lower respiratory disease	2.33 (1.16 to 3.52)	2.57 (1.17 to 3.99)	1.90 (–0.04 to 3.87)	2.38 (–0.14 to 4.96)	2.32 (1.02 to 3.63)
Chronic obstructive pulmonary disease	2.33 (1.12 to 3.56)	2.47 (1.03 to 3.93)	2.10 (0.08 to 4.16)	2.71 (0.01 to 5.48)	2.25 (0.93 to 3.58)
Other respiratory disease	1.52 (–0.69 to 3.77)	1.31 (–1.81 to 4.53)	1.68 (–1.41 to 4.87)	3.19 (–1.64 to 8.27)	1.09 (–1.34 to 3.59)
Digestive diseases	–0.09 (–1.71 to 1.55)	–1.18 (–3.26 to 0.94)	1.61 (–1.07 to 4.35)	–0.71 (–3.19 to 1.83)	0.42 (–1.73 to 2.63)
Esophagus, stomach, and duodenum disease	0.34 (–3.10 to 3.89)	–0.95 (–5.55 to 3.89)	1.90 (–3.14 to 7.21)	1.25 (–5.01 to 7.93)	–0.03 (–4.08 to 4.20)
Liver disease	0.28 (–2.63 to 3.27)	–0.42 (–3.71 to 2.98)	2.44 (–3.58 to 8.84)	–1.34 (–4.64 to 2.08)	5.14 (–0.77 to 11.39)
Other digestive disease	–0.07 (–3.72 to 3.71)	–1.16 (–5.95 to 3.88)	1.39 (–4.29 to 7.41)	0.49 (–6.45 to 7.94)	–0.34 (–4.55 to 4.06)
Nervous diseases	0.32 (–2.78 to 3.51)	0.03 (–4.20 to 4.44)	0.64 (–3.93 to 5.42)	1.18 (–2.92 to 5.46)	–0.95 (–5.54 to 3.86)
Genitourinary diseases	0.57 (–2.04 to 3.24)	1.12 (–2.31 to 4.67)	–0.07 (–3.97 to 3.98)	–0.29 (–4.12 to 3.71)	1.17 (–2.35 to 4.81)
Urinary diseases	0.57 (–2.04 to 3.24)	1.12 (–2.31 to 4.67)	–0.07 (–3.97 to 3.98)	–0.29 (–4.12 to 3.71)	1.17 (–2.35 to 4.81)
Renal failure	0.82 (–2.75 to 4.52)	2.22 (–2.51 to 7.16)	–0.87 (–6.17 to 4.74)	–0.20 (–5.41 to 5.30)	1.68 (–3.08 to 6.67)
External causes	1.32 (–0.01 to 2.67)	1.36 (–0.37 to 3.11)	1.26 (–0.76 to 3.33)	1.38 (–0.33 to 3.12)	1.27 (–0.80 to 3.38)
Road traffic injury	0.28 (–2.89 to 3.55)	0.68 (–2.85 to 4.34)	–1.10 (–7.86 to 6.17)	–0.40 (–3.66 to 2.97)	13.02 (–0.64 to 28.55)
Intentional self-harm	3.99 (0.22 to 7.90)	2.08 (–2.59 to 6.97)	7.09 (1.06 to 13.48)	3.05 (–0.93 to 7.20)	9.85 (–0.31 to 21.03)
Endocrine diseases	2.40 (0.84 to 3.98)	1.76 (–0.48 to 4.06)	2.95 (0.81 to 5.14)	1.01 (–1.36 to 3.43)	3.41 (1.35 to 5.51)
Diabetes	2.04 (0.12 to 3.99)	1.15 (–1.54 to 3.91)	2.97 (0.25 to 5.76)	1.02 (–1.72 to 3.83)	3.00 (0.31 to 5.77)
Neoplasm	1.18 (0.59 to 1.78)	1.03 (0.28 to 1.77)	1.45 (0.48 to 2.42)	1.06 (0.34 to 1.79)	1.41 (0.41 to 2.42)
Pancreas disease	0.22 (–3.12 to 3.69)	2.35 (–2.30 to 7.24)	–2.11 (–6.76 to 2.78)	0.29 (–3.86 to 4.61)	0.24 (–5.18 to 5.96)

Among the 26 disease subcategories, after NO_2_ exposure, there were significant increases in the top 10 causes with the highest risk of mortality: intentional self-harm (3.99%, 95% CI 0.22%-7.90%), hypertensive diseases (3.39%, 95% CI 1.80%-4.99%), ischemic stroke (2.84%, 95% CI 1.35%-4.35%), cerebrovascular diseases (2.48%, 95% CI 1.70%-3.27%), stroke (2.48%, 95% CI 1.55%-3.42%), influenza and pneumonia (2.47%, 95% CI 1.35%-3.60%), chronic obstructive pulmonary disease (2.33%, 95% CI 1.12%-3.56%), chronic lower respiratory disease (2.33%, 95% CI 1.16%-3.52%), chronic rheumatic heart disease (2.32%, 95% CI 1.56%-3.08%), and diabetes (2.04%, 95% CI 0.12%-3.99%; Table 3 and Figure S3 in Multimedia Appendix 1).

The results of the stratified analysis are summarized in Table 3. For all-cause mortality, the impact of NO_2_ was relatively higher among women than men, but the difference was not statistically significant (P=.32). Older adults were at a higher risk than the younger age groups (P=.02), and their death rates due to NO_2_ exposure increased by 2.15% (95% CI 1.68%-2.62%) and 1.15% (95% CI 0.66%-1.63%), respectively. Age was a particularly significant factor for deaths due to nonaccidental causes and cardiovascular diseases.

Stratification analyses by meteorological factor levels were further conducted (Table 4). We observed relatively higher effect estimates of NO_2_ on mortality for low levels of temperature, relative humidity, and wind speed than high levels, although most of the differences between the subgroups were not statistically significant. For air pressure, relatively higher effect estimates were found for all causes, nonaccidental causes, cardiovascular diseases, respiratory diseases, and neoplasms at low levels of air pressure than at high levels of air pressure. However, the opposite was true for endocrine diseases.

**Table 4:** Stratified analysis of the percent increase in mortality as a result of different diseases per 10 μg/m^3^ increase in NO_2_ at a lag of 0 to 4 days during 2010 to 2018 in Guangzhou, China.

Causes	Total, % change (95% CI)	Temperature, % change (95% CI)				Humidity, % change (95% CI)				Wind speed, % change (95% CI)				Air pressure, % change (95% CI)		
		Low	High	*P* value	Low		High	*P* value	Low		High	*P* value	Low		High	*P* value
All causes	1.73 (1.36 to 2.09)	2.14 (1.71 to 2.57)	1.42 (0.53 to 2.33)	.16	1.95 (1.45 to 2.45)		1.41 (0.85 to 1.97)	.16	1.70 (1.20 to 2.21)		1.65 (1.06 to 2.24)	.89	1.78 (0.91 to 2.65)		2.19 (1.75 to 2.63)	.41
Nonaccidental causes	1.75 (1.38 to 2.12)	2.21 (1.77 to 2.65)	1.28 (0.37 to 2.20)	.07	2.01 (1.50 to 2.53)		1.37 (0.80 to 1.94)	.10	1.71 (1.19 to 2.23)		1.70 (1.10 to 2.31)	.99	1.74 (0.84 to 2.64)		2.24 (1.80 to 2.69)	.32
Cardiovascular diseases	2.06 (1.54 to 2.59)	2.63 (2.01 to 3.24)	1.79 (0.45 to 3.15)	.27	2.44 (1.73 to 3.16)		1.31 (0.51 to 2.12)	.04	2.14 (1.40 to 2.88)		1.77 (0.95 to 2.60)	.51	2.10 (0.82 to 3.41)		2.86 (2.23 to 3.49)	.31
Respiratory diseases	2.32 (1.51 to 3.13)	2.88 (1.90 to 3.86)	2.68 (0.58 to 4.83)	.87	2.58 (1.46 to 3.71)		1.93 (0.69 to 3.18)	.45	2.53 (1.42 to 3.64)		2.15 (0.82 to 3.51)	.67	2.45 (0.52 to 4.41)		2.97 (1.96 to 4.00)	.64
Endocrine diseases	2.40 (0.84 to 3.98)	3.04 (1.15 to 4.97)	0.88 (–3.15 to 5.07)	.35	2.63 (0.43 to 4.87)		1.82 (–0.50 to 4.19)	.62	1.94 (–0.20 to 4.13)		2.99 (0.46 to 5.59)	.54	4.57 (0.68 to 8.62)		2.59 (0.68 to 4.54)	.38
Neoplasms	1.18 (0.59 to 1.78)	1.38 (0.66 to 2.09)	0.27 (–1.16 to 1.73)	.18	1.38 (0.56 to 2.21)		1.05 (0.13 to 1.97)	.60	1.19 (0.38 to 2.02)		0.88 (–0.08 to 1.85)	.63	0.90 (–0.46 to 2.28)		1.10 (0.36 to 1.83)	.81

The sensitivity analysis showed that each of the different modeling strategies provided roughly similar results. First, the calendar date and weather variables were varied using 3 to 7 *df*s and 3 to 6 *df*s, respectively, year-on-year. Second, we used DLNM to adjust for temperature and found that the estimated effect of NO_2_ was slightly attenuated but still statistically significant. Third, the main findings were stable with additional adjustment for SO_2_, PM_2.5_, O_3_, PM_10_, and CO in the 2-pollutant model. As shown in Figure S5 in Multimedia Appendix 1, the model demonstrated stability and reliability after using alternative *df*s to control for the meteorological confounders.

## Discussion

### Principal Findings

To the best of our knowledge, this is one of few studies to investigate the relationship between NO_2_ exposure and deaths due to multiple causes. An increment of 10 μg/m^3^ in NO_2_ increased the risk of all-cause mortality by 1.73% and had a similar impact on deaths due to nonaccidental causes (1.75% increase), cardiovascular diseases (2.06% increase), respiratory diseases (2.32% increase), endocrine diseases (2.40% increase), and neoplasms (1.18% increase). Following NO_2_ exposure, older adults were at a higher risk of death due to all causes, nonaccidental causes, and cardiovascular diseases. Furthermore, the impact of NO_2_ was higher in warm temperatures than in cold temperatures.

Multiple studies have found that low wind speeds lead to high atmospheric stability and high levels of air pollution, which in turn have led to higher numbers of COVID-19–related infections and deaths [29,30]. Atmospheric stability based on low wind speeds reduces the diffusion of gases and air pollution particles [29]. The results of our analyses were similar. However, several studies found that wind speed and COVID-19 are positively correlated. An inverted U-shaped dose-response curve was found for wind speed and COVID-19 [30]. A Japanese study involving 74 participants reported that low humidity and high air pressure contribute to brain hemorrhage [31]. In contrast, a hospital-based study in Mexico showed no significant relationship between barometric pressure and stroke [32]. However, the influence of humidity and air pressure may be not as strong as other weather conditions such as temperature. Therefore, we included humidity, wind speed, and air pressure as confounding factors in the model to avoid potential bias.

Previous studies on the association between NO_2_ and mortality mainly focused on overall causes, cardiovascular diseases, and respiratory diseases [13,15,16]. Our study also showed a statistically significant association between NO_2_ and endocrine system diseases. We found that the impact of NO_2_ exposure on mortality rates associated with endocrine, respiratory, and cardiovascular diseases was higher than that reported in a previous meta-analysis [33]. Studies previously conducted in China also reported that, for every 10 μg/m^3^ increase in NO_2_ concentration, mortality rates related to all causes, endocrine diseases, cardiovascular diseases, and respiratory diseases increased by 0.96%, 1.13%, 1.01%, and 1.22%, respectively [34,35]. In western countries, available data indicate that NO_2_ exposure leads to an increase in total mortality as well as mortality associated with endocrine diseases, cardiovascular diseases, and respiratory diseases, by 0.33%, 0.38%, 0.40%, and 0.38%, respectively [13,36]. These differences could be attributed to racial factors and the varying analytical techniques among the different studies.

NO_2_ can affect disease-related mortality through various mechanisms. For instance, altered immune responses and inflammatory reactions following NO_2_ exposure may lead to endocrinological disorders. Inhaled NO_2_ triggers production of proinflammatory cytokines by alveolar macrophages, resulting in local oxidative stress. Furthermore, the cytokines enter systemic circulation and affect distant tissues, contributing to autoimmune responses and metabolic dysfunction [37,38]. NO_2_ is also a respiratory irritant that generates highly reactive, free radicals [39], which can cause severe lung injury, and death can occur depending on the dose and duration of exposure [40]. In addition, for individuals exposed to NO_2_, levels of inflammatory markers such as IL-12 and C-reactive protein in the blood are elevated, which may destabilize atherosclerotic plaques and result in their rupture, leading to increased blood pressure and enhanced thrombosis formation [41].

Gender and age affect the level of sensitivity to air pollution [42,43]. The impact of the aforementioned factors on the effects generated by NO_2_ were largely inconclusive in previous studies. We found that gender was not a determinant of mortality risk due to NO_2_ exposure, which is consistent with previous studies [15,44-46]. However, one study showed that men are more susceptible to the detrimental effects of NO_2_ [14], which can be attributed to occupational and physiological differences. Furthermore, several studies have shown that older adults are predisposed to NO_2_-related mortality risk [13,15,23,47]. Consistent with this, the older adults in our cohort showed a higher risk of death due to all causes, nonaccidental causes, and cardiovascular diseases following NO_2_ exposure. A possible explanation is that older people have higher rates of chronic diseases and weaker immune systems, which may exacerbate the pathological effects of environmental NO_2_ pollution [48]. However, one study showed a link between NO_2_ exposure and the first episode of atrial fibrillation in young adults but not older adults [49]. NO_2_ is a significant risk factor for sudden death and melancholia among younger individuals, which may explain these results. Considering the rapidly aging population and changes in disease patterns, public policymaking, risk assessment, and air pollution standards should be modified to reduce the impact of NO_2_ on public health in China.

The highest risk of death due to NO_2_ exposure was observed at a lag of 1 or 2 days and persisted for 4 days. However, some studies have observed the strongest link between NO_2_ levels and the number of hospital admissions on day 0 [46]. Similar trends in the effects of a lag of NO_2_ exposure have been reported for the mortality due to all causes, cardiovascular diseases, and respiratory diseases [13]. Moreover, the shape of the lag effects can be described by 2 different patterns. The first is that the NO_2_ levels recorded on the previous 2 days have a greater effect on mortality due to all causes, nonaccidental causes, circulatory diseases, respiratory diseases, endocrine diseases, and tumors. The second is that mortality resulting from digestive, nervous, and urogenital diseases as well as external systems tends to be more evenly distributed during the first 6 days. These inconsistencies may be a result of differences in the biological mechanisms that underlie the biological effects of NO_2_.

We also found that the impact of NO_2_ exposure was higher on cold days, which is consistent with the findings of previous studies. For instance, studies conducted in Shanghai and Wuhan have shown that the effects of NO_2_ are highest in the winter [50,51]. Moreover, other research groups in China reported no substantial variation in the effects of NO_2_ on mortality due to nonaccidental causes and cardiovascular diseases across different seasons [23,52,53]. However, a large time series analysis conducted in the northeast United States reported a peak mortality risk from NO_2_ exposure in the summer [54]. Likewise, an Italian study conducted between April and September also found a stronger association between NO_2_ and mortality due to natural causes, cardiac diseases, and respiratory diseases [14]. A meta-analysis further demonstrated that NO_2_ had a greater impact on hospital admissions and mortality in hot weather (May 1 to September 30) [55]. One possible explanation is that people are exposed to higher levels of NO_2_ during the warmer periods of the year due to greater involvement in outdoor activities [14]. Furthermore, the production of NO_2_ also increases at higher temperatures [56]. Another reason is that heat may promote thrombosis by increasing blood viscosity and secondary cholesterol levels [57]. Finally, individual susceptibility to air pollutants may increase in the summer [58]. These inconsistent results may be due to the influence of concentrations of NO_2_ components, regional gaseous pollutants, climatic conditions, resident exposure patterns, socioeconomic characteristics, and different data analysis methods.

This study has several salient points. First, the study showed that NO_2_ was related not only to all-cause mortality but also to cause-specific mortality. Second, the relationship between NO_2_ and mortality due to different causes and the potential modifying effects of gender, age, and season were analyzed. These findings may have important implications for developing targeted strategies to protect vulnerable populations from the harmful effect of NO_2_. The detrimental effects of NO_2_ can be minimized by reducing time spent outdoors and physical exertion, wearing a mask when going out, avoiding heavily trafficked roads, and increasing immunity [59].

Nevertheless, there are several limitations that need to be considered. First, our study was limited to the Guangzhou area, and the results cannot be generalized to other regions due to differences in the population structure and environmental characteristics. Second, environmental monitoring data may only indicate population averages rather than individual levels, which may have introduced inevitable measurement bias [60]. Third, the classification of the cause of death was based only on ICD-10 codes and does not detail the actual cause of death of the patient, which may also lead to bias. Finally, causal relationships and the underlying pathophysiology of our findings could not be ascertained and warrant further research.

### Conclusions

In summary, our study comprehensively examined the association between NO_2_ exposure and a spectrum of causes for the risk of mortality. Through subgroup analysis, we found that gender did not significantly modify the NO_2_-related mortality risks, while older adults were more susceptible to death due to all causes, nonaccidental causes, and cardiovascular diseases when exposed to NO_2_. Furthermore, the association between NO_2_ and cause-specific mortality is stronger in the warm season. We found a relatively higher effect of NO_2_ on health on days with higher temperatures, relative humidity, and wind speed but lower pressure levels, though the between-group differences were not statistically significant. Considering heterogeneity in socioeconomic characteristics and population structure among different regions, as well as the limitations of the inaccuracy of monitoring data and measurement error of ICD classifications, our findings still need to be confirmed in other regions. Our study provides new evidence to develop prevention-oriented health policies, highlighting that it is necessary to strengthen air quality standards to protect public health from NO_2_ pollution.

## Appendix

Multimedia Appendix 1 Supplementary tables and figures.

## Abbreviations

DLNM: distributed lag nonlinear model
ICD-10: 10th Revision of the International Classification of Diseases
NS: natural cubic spline function
PM: particulate matter
