# Supplementary material for: Association Between Nitrogen Dioxide Pollution and Cause-Specific Mortality in China: Cross-Sectional Time Series Study
Source: JMIR Public Health Surveill. 2024 Feb 5;10:e44648. doi: 10.2196/44648 (PMC10877496; doi:10.2196/44648)
Supplement: Multimedia Appendix 1 [file publichealth_v10i1e44648_app1.docx]

**Table of contents**

| **Title** | **Page** |
| --- | --- |
| **Table S1.** Summary statistics of mortality as a result of different diseases on number of deaths during 2010-2018 in Guangzhou. | 2 |
| **Table S2.** Results of generalized Poisson regression. | 3 |
| **Table S3.** Summary main results for our study and other studies. | 4 |
| **Figure S1.** Correlations (Spearman correlation coefficient) between air pollution and weather conditions. (Note: *: P < 0.05; **: P < 0.01; ***: P < 0.001). | 5 |
| **Figure S2.** The relative risk of death resulting from the different causes associated with an 10 μg/m^3^ increase in NO_2_ levels with a one-day delay (lag days of 0,1,2, ..., to 6) for each of the different genders and age groups. | 6 |
| **Figure S3.** Percentage increase (95%CI) in mortality as a result of different diseases due to NO_2_ exposure with a lag of 0-4 days. | 7 |
| **Figure S4.** The dose-effect curves of NO_2_ and mortality as a result of different diseases with a lag of 0 days in Guangzhou, China. | 8 |
| **Figure S5.** The sensitivity analyses of the cumulative relative risk (95% CI) of mortality associated with an 10 μg/m^3^ increase in NO_2_ with a lag of 0-4 days. | 9 |

**Table S1.** Summary statistics of mortality as a result of different diseases on number of deaths during 2010-2018 in Guangzhou.

| Diseases | ICD-10 | Number of deaths | |
| --- | --- | --- | --- |
|  |  | Total number during 2010-2018 | Daily mean (range) |
| **All-cause** | A00-Z99 | 413703 | 126 (53, 251) |
| **Non-accidental** | A00-R99 | 391543 | 119 (47, 238) |
| **Cardiovascular disease** | I00-I99 | 160226 | 49 (13, 115) |
| Chronic rheumatic heart diseases | I05-I09 | 69052 | 21 (2, 50) |
| Hypertensive diseases | I10-I15 | 15491 | 5 (0, 19) |
| Ischemic heart disease | I20-I25 | 66105 | 20 (3, 50) |
| Acute ischemic heart disease | I20-I22, I24 | 32051 | 10 (0, 27) |
| Acute myocardial infarction | I21-I22 | 29986 | 9 (0, 25) |
| Myocardial infarction | I21-I23 | 29992 | 9 (0, 25) |
| Chronic ischemic heart disease | I25 | 34048 | 10 (0, 32) |
| Other forms of heart disease | I30-I52 | 5817 | 2 (0, 8) |
| Cerebrovascular | I60-I69 | 65565 | 20 (2, 49) |
| Stroke | I60-I64 | 43447 | 13 (0, 33) |
| Intracerebral hemorrhagic stroke | I61 | 15904 | 5 (0, 17) |
| Ischemic stroke | I63 | 17203 | 5 (0, 17) |
| Arteries, arterioles and capillaries | I70-I79 | 2149 | 1 (0, 6) |
| **Respiratory disease** | J00-J99 | 63290 | 19 (5, 51) |
| Influenza and pneumonia | J09-J18 | 31655 | 10 (0, 29) |
| Chronic lower respiratory | J40-J47 | 30240 | 9 (0, 31) |
| Chronic obstructive pulmonary disease | J40-J44 | 28403 | 9 (0, 30) |
| Other respiratory | J95-J99 | 7636 | 2 (0, 13) |
| **Digestive disease** | K00-K93 | 12787 | 4 (0, 12) |
| Oesophagus, stomach and duodenum | K20-K31 | 2939 | 1 (0, 5) |
| Liver | K70-K77 | 4202 | 1 (0, 7) |
| Other digestive | K90-K93 | 2376 | 1 (0, 5) |
| **Nervous disease** | G00-G99 | 3659 | 1 (0, 7) |
| **Genitourinary disease** | N00-N99 | 5314 | 2 (0, 8) |
| Urinary | N00-N39 | 5314 | 2 (0, 8) |
| Renal failure | N17-N19 | 2681 | 1 (0, 7) |
| **External causes** | V01-Y89 | 22147 | 7 (0, 19) |
| Road traffic injury | V01-V89 | 3864 | 1 (0, 7) |
| Intentional self-harm | X60-X84 | 2947 | 1 (0, 7) |
| **Endocrine diseases** | D50-D89, E00-E90 | 14972 | 5 (0, 16) |
| Diabetes | E10-E14 | 9642 | 3 (0, 12) |
| **Neoplasms** | C00-D48 | 115703 | 35 (12, 68) |
| Pancreas | C25 | 3376 | 1 (0, 6) |

**Table S2.** Results of generalized Poisson regression.

| Parametric coefficients | | | | |
| --- | --- | --- | --- | --- |
| Environmental variables | Estimate | Std. Error | t value | Pr(>\|t\|) |
| Constant | 3.547241 | 0.010663 | 332.682 | .001*** |
| Holiday | -0.006200 | 0.012271 | -0.505 | .613 |
| Dow | -0.001939 | 0.001529 | -1.268 | .205 |
| NO_2_ | 0.002121 | 0.001952 | 1.086 | .277 |
| Approximate significance of smooth terms | | | | |
|  | Edf | Ref.df | F | p-value |
| s(trend) | 62 | 62 | 27.454 | .001*** |
| s(humidity) | 3 | 3 | 5.737 | .001*** |
| s(temperature) | 6 | 6 | 1.732 | .110 |
| s(air pressure) | 3 | 3 | 2.072 | .100 |
| s(wind speed) | 3 | 3 | 6.294 | .001*** |

Note: *: P < 0.05; **: P < 0.01; ***: P < 0.001

Goodness of fit index: R^2^(adj)=0.69.

**Table S3.** Summary main results for our study and other studies.

| **Category** | **Our study** | **Other study** |
| --- | --- | --- |
| Percentage increase (95%CI) in mortality as a result of different diseases per 10 μg/m^3^ increase in NO_2_ | 1. 1.73% in all-cause 2. 1.75% in non-accidental 3. 2.06% in cardiovascular 4. 2.32% in respiratory 5. 2.40% in endocrine 6. 1.18% in neoplasms | **In China:**   1. 0.96% in all-cause 2. 1.13% in endocrine 3. 1.01% in cardiovascular 4. 1.22% in respiratory   **In Western:**   1. 0.33% in all-cause 2. 0.38% in endocrine 3. 0.40% in cardiovascular 4. 0.38% in respiratory |
| Meteorological effect:  temperature  relative humidity  wind speed  air pressure | 1. low levels of temperature, relative humidity, wind speed and air pressure lead to higher effect estimates of NO_2_ on All-cause, Non-accidental, Cardiovascular, Respiratory, Endocrine, and Neoplasm 2. low level of air pressure lead to relatively higher effect estimates for all-cause, non-accidental, cardiovascular, respiratory and neoplasm, but opposite trend for endocrine 3. higher effect in cold day | 1. low wind speeds lead to high atmospheric stability and high levels of air pollution 2. low wind speeds lead to higher numbers of COVID-19-related infections and deaths 3. low humidity and high air pressure are prone to brain hemorrhage in Japanese 4. no significant relationship between barometric pressure and stroke in Mexico 5. higher effect in cold day in Shanghai and Wuhan 6. higher effect in hot day in USA, Italian |
| Modification effect:  gender  age | 1. non-significant in gender 2. higher risk of death due to all-causes, non-accidental and cardiovascular in elderly | 1. males are more susceptible 2. higher risk of death due to atrial fibrillation, sudden death and melancholia in young adults |
| Lag effect of NO_2_ | 1. highest risk at the lag of 1 or lag 2 days, and persisted to last for 4 days for death mortality | 1. similar lagged effects trend for all causes, cardiovascular and respiratory 2. on the previous two days have a greater effect on all-cause, non-accident, circulatory, respiratory, endocrine, and tumor caused mortality 3. more evenly distributed during the first 6 days on digestive, nervous, urogenital, and external system related causes |


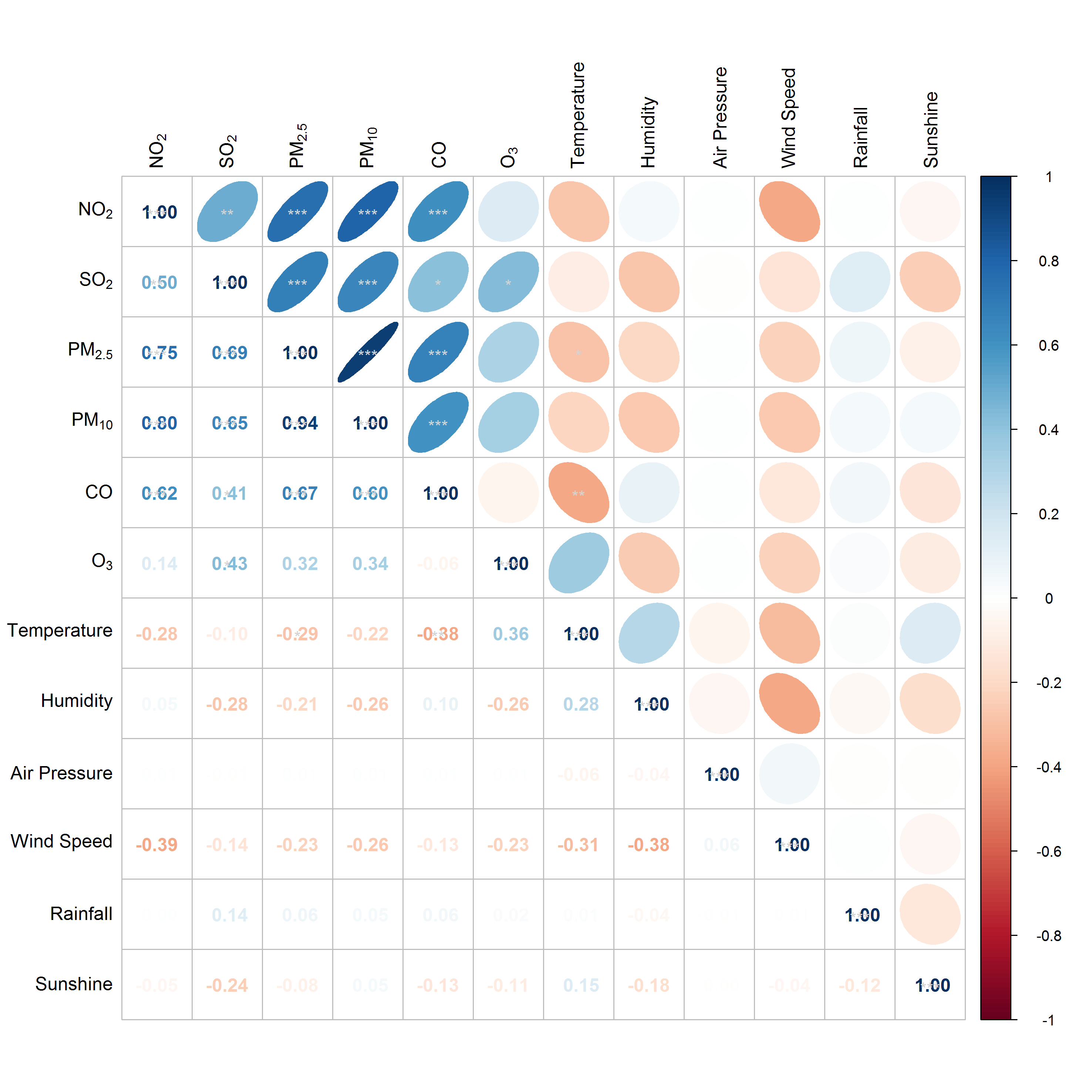


**Figure S1.** Correlations (Spearman correlation coefficient) between air pollution and weather conditions. (Note: *: P < 0.05; **: P < 0.01; ***: P < 0.001).


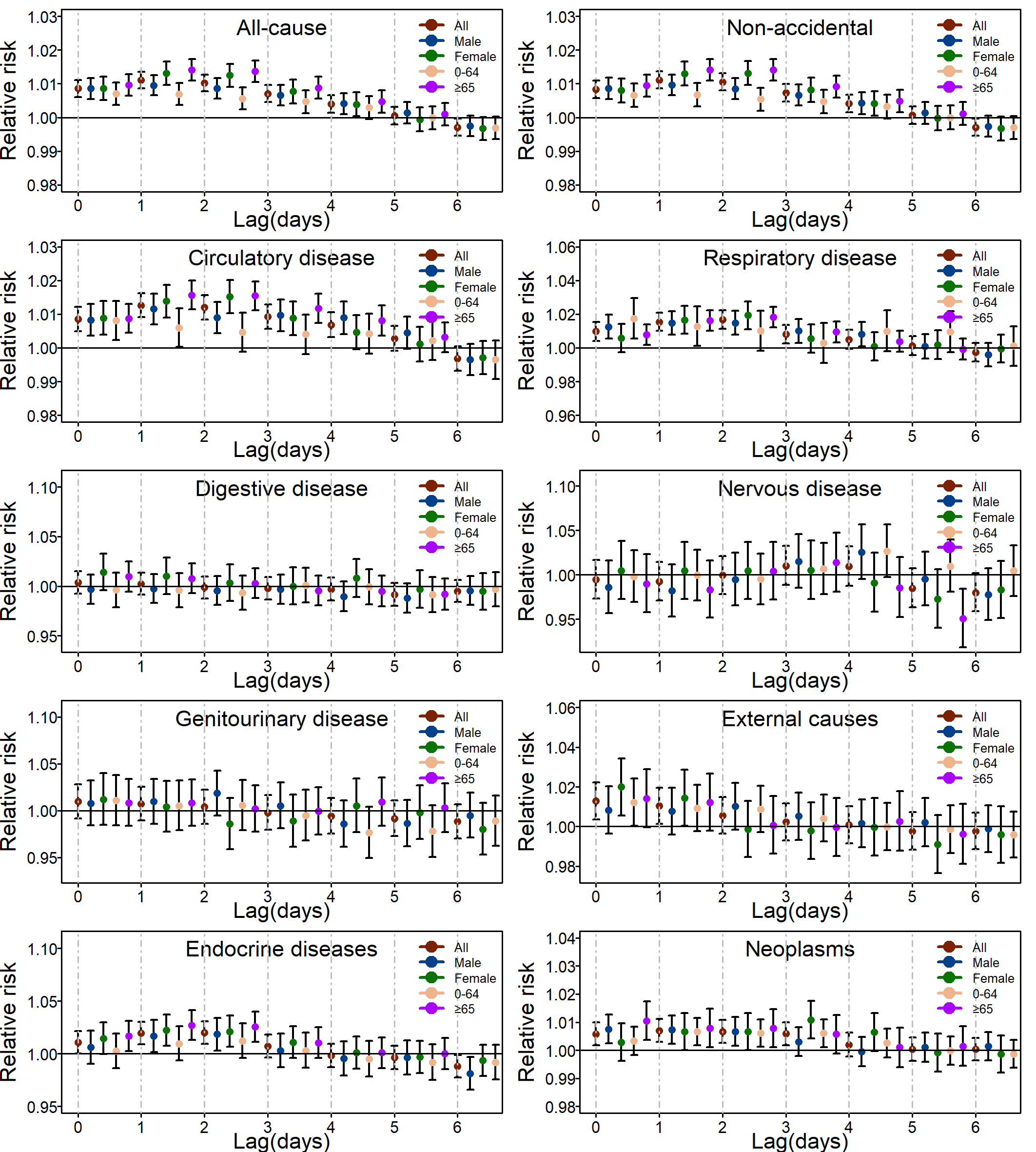


**Figure S2.** The relative risk of death resulting from the different causes associated with an 10 μg/m^3^ increase in NO_2_ levels with a one-day delay (lag days of 0,1,2, ..., to 6) for each of the different genders and age groups.


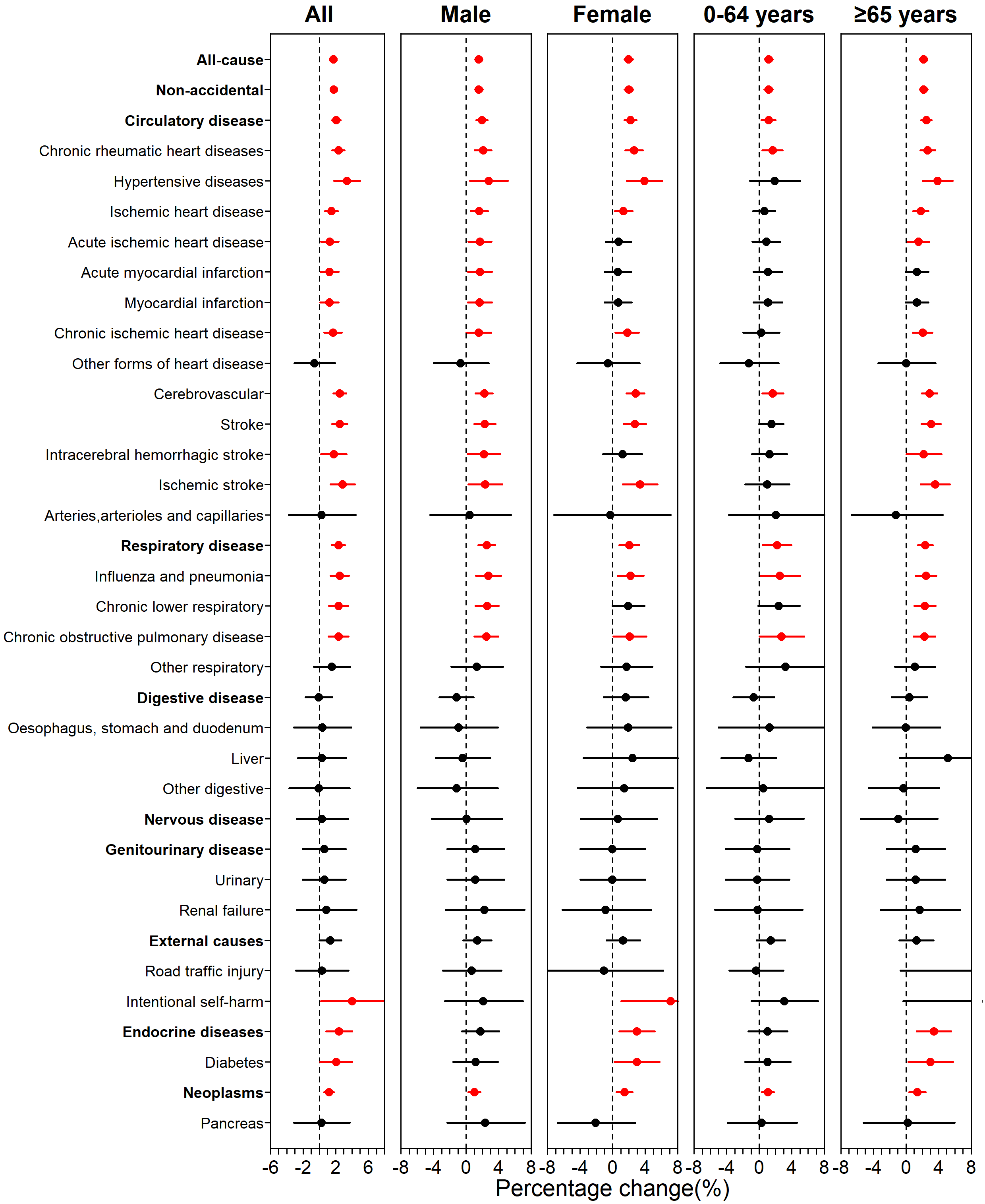


**Figure S3.** Percentage increase (95%CI) in mortality as a result of different diseases due to NO_2_ exposure with a lag of 0-4 days. The point estimates and 95% confidence intervals were used as statistical estimates. The red lines indicate results that are statistically significant, while black lines indicate results that are not statistically significant.


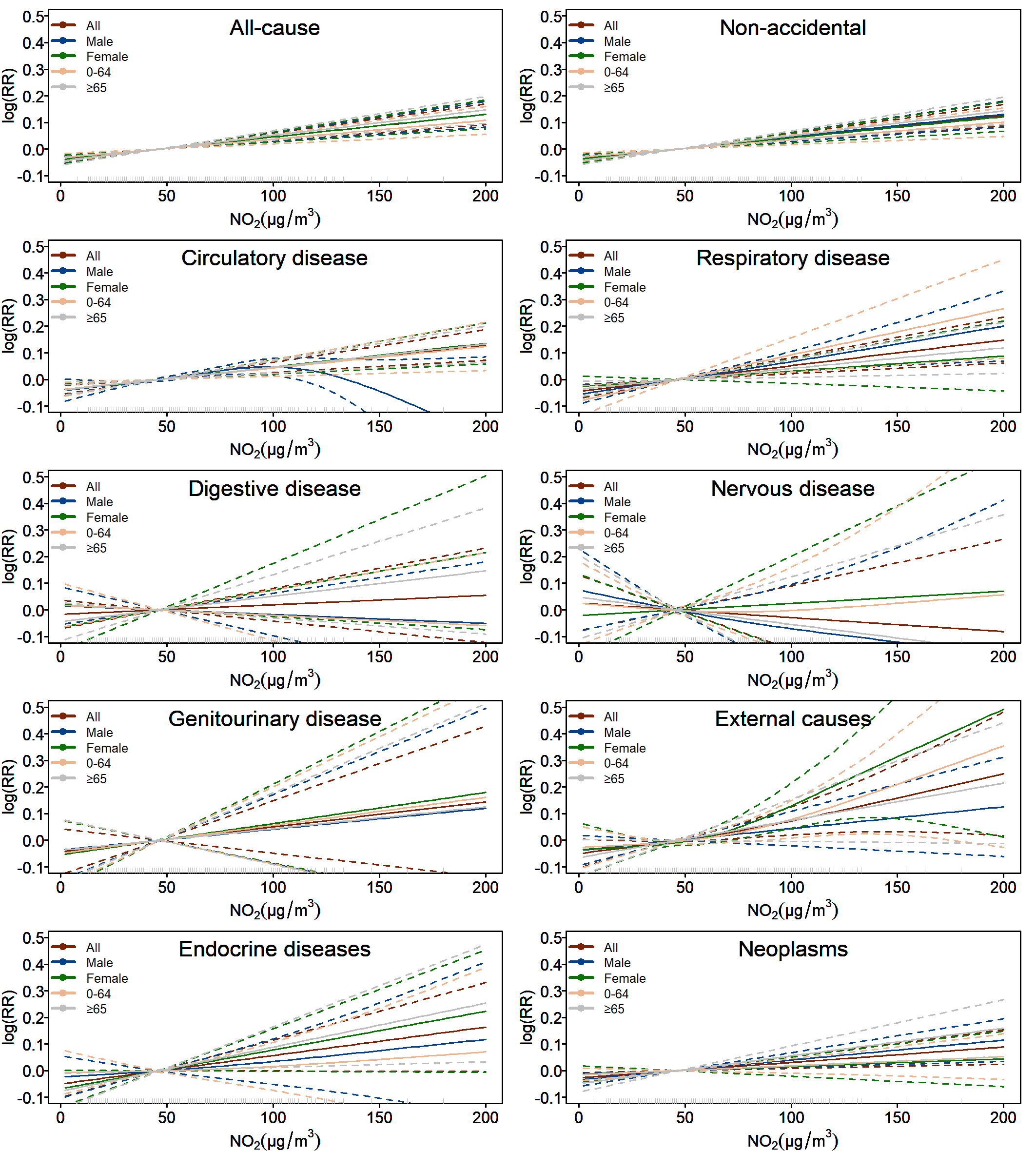


**Figure S4.** The dose-effect curves of NO_2_ and mortality as a result of different diseases with a lag of 0 days in Guangzhou, China.


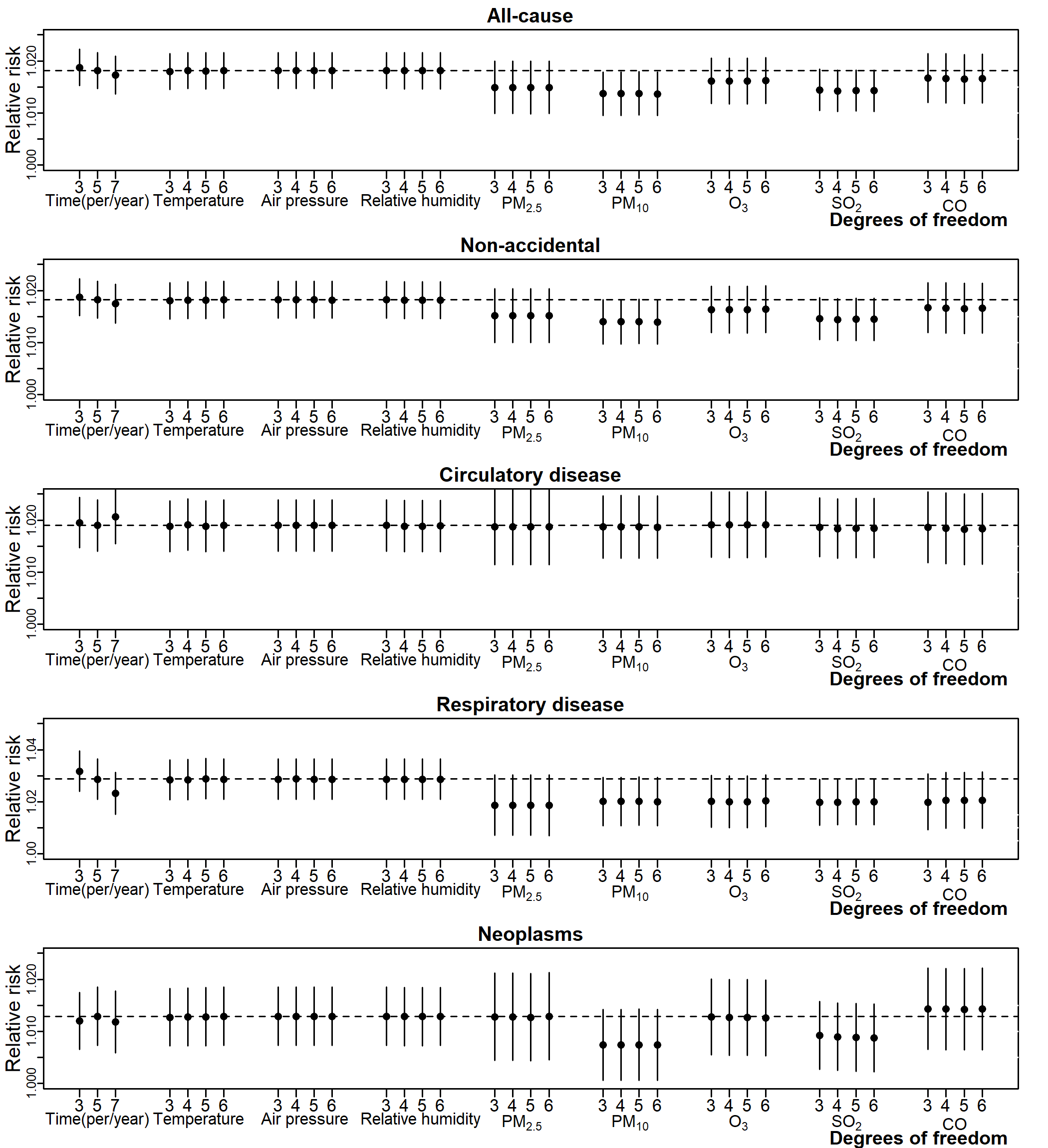


**Figure S5.** The sensitivity analyses of the cumulative relative risk (95%CI) of mortality associated with an 10 μg/m^3^ increase in NO_2_ with a lag of 0-4 days. The dashed lines denote the relative risk of NO_2_ on mortality based on the main model.
